# Predictors of 30-Day Readmission Following Cervical Laminoplasty in 3,085 Patients: An American College of Surgeons National Surgical Quality Improvement Program (ACS-NSQIP) Database Study

**DOI:** 10.7759/cureus.32480

**Published:** 2022-12-13

**Authors:** Jason Kim, Simon G Ammanuel, Paul S Page, Darnell T Josiah

**Affiliations:** 1 Neurological Surgery, University of Wisconsin School of Medicine and Public Health, Madison, USA; 2 Neurological Surgery, University of Wisconsin Hospitals and Clinics, Madison, USA; 3 Neurosurgery, University of Wisconsin, Madison, USA

**Keywords:** nsqip, readmission, elderly, laminoplasty, cervical spine

## Abstract

Background

Cervical laminoplasty is a surgical alternative to laminectomy and fusion for treating multi-level cervical spondylotic myelopathy. There is limited evidence evaluating readmission incidence and risk factors following cervical laminoplasty. Here, we provide a retrospective review evaluating preoperative risk for 30-day readmission following cervical laminoplasty.

Methodology

The American College of Surgeons National Surgical Quality Improvement Program (ACS-NSQIP) database was used to identify patients undergoing laminoplasty as defined by Current Procedural Terminology codes 63050 and 63051. Patients were then categorized based on whether 30-day readmission occurred, and preoperative risk factors were examined. Continuous and categorical variables were analyzed using Student’s t-test or Fisher’s exact test. Multivariate regression analysis was performed for each variable, with p-values of <0.05 considered significant.

Results

In total, 3,085 patients were identified as undergoing posterior cervical laminoplasty. Of these, 2,938 patients did not require readmission, and 147 patients were readmitted, representing a 4.77% 30-day readmission rate. For all patients, sepsis (odds ratio (OR) = 5.58, p = 0.03), dialysis (OR = 3.46, p = 0.01), American Society of Anesthesiologists class >2 (OR = 1.69, p = 0.011), and hypertension (OR = 1.51, p = 0.04) were predictive of readmission. A subgroup analysis was performed for all geriatric patients (aged >65). In total, 1,353 patients were identified, of whom 76 were readmitted, demonstrating a readmission rate of 5.62%. For the elderly patients, hypertension (OR = 1.98, confidence interval (CI) = 1.04-3.75, p = 0.04) and independent status (OR = 0.39, CI = 0.21-0.74, p = 0.004) were predictive of readmission.

Conclusions

Assessment of predictors for readmission is important for patient education and setting appropriate clinical expectations for surgeons and providers. Preoperative hypertension, dialysis, and sepsis were risk factors for 30-day readmission following cervical laminoplasty, with functional status being a unique risk factor for elderly patients.

## Introduction

Cervical laminoplasty is a surgical option to provide decompression in the setting of multi-level cervical spondylotic myelopathy [1-3]. Laminoplasty reduces the risk of postoperative kyphosis and preserves segmental motion [3,4]. Readmissions contribute significantly to the financial burden of hospital systems within the United States and have been a long-standing quality improvement target for many healthcare organizations. This is primarily due to the hospital readmission reduction program under the Affordable Care Act that allows the Centers for Medicare and Medicaid Services to inflict penalties on hospitals that do not meet specific benchmark standards for readmissions and other factors [5]. Given this, readmission patterns and preoperative risk factors are essential to identify to minimize readmission in high-risk populations [6-8].

Several studies have evaluated risk factors for readmission following spine surgery, including lumbar decompression and fusions and anterior cervical discectomy and fusions. Despite this, these studies are relatively heterogeneous and not specific to cervical laminoplasty. Additionally, some studies have compared readmission and complication rates following cervical laminectomy and fusion in comparison with laminoplasty. However, these studies did not evaluate risk factors for readmission in laminoplasty patients [1]. Here, we utilize the American College of Surgeons National Surgical Quality Improvement Program (ACS-NSQIP) database to report the largest study to date characterizing risk factors for 30-day readmission following cervical laminoplasty.

## Materials and methods

The ACS-NSQIP database is a multi-hospital-level database that contains many preoperative, intraoperative, and postoperative variables from patients across 250 urban and rural hospitals in the United States. The database utilizes random sampling of patients within participating hospitals that have obtained approval from their respective institutional review boards, resulting in standardized data with interrater reliability and de-identified patient data that does not require patient consent [9]. A retrospective analysis of the ACS-NSQIP database was performed for all surgical patients who underwent cervical laminoplasty, Current Procedural Terminology code 63050 and 63051, between 2011 and 2020. Patients were categorized based on their status of readmission. Risk factors for readmission were then evaluated, including body mass index (BMI), operation time, the total length of stay, preoperative duration, sex, race (White, Black, Asian, Native American, other), geriatric age (≥65 years), home discharge, elective surgery status, diabetes, smoking status, dyspnea, independent status, chronic obstructive pulmonary disease (COPD), congestive heart failure (CHF), hypertension, renal failure, dialysis, cancer, chronic steroid use, sepsis/septic shock, clean wound class, and American Society of Anesthesiologist (ASA) class >2.

Statistical analyses were performed using SPSS version 28 (IBM Corp., Armonk, NY, USA). Analysis was performed between the non-readmitted and readmitted groups. Continuous and categorical variables were initially analyzed with either Student’s t-test or Fisher’s exact test. Subsequently, a multivariate regression analysis was performed for each variable. Statistical significance was deemed at p-values of <0.05 for each test. Odds ratios (ORs) were reported for regression analysis with 95% confidence intervals (CIs).

## Results

In total, 3,085 patients were identified as undergoing posterior cervical laminoplasty. Of these, 2,938 patients did not require readmission, and 147 patients were readmitted, representing a 4.77% 30-day readmission rate. There were no statistically significant differences in baseline demographics of readmitted patients (p > 0.05) (Table 1). Preoperative comorbidities, independent status, hypertension, dialysis dependence, sepsis or septic shock, non-clean wound class, and ASA class >2 demonstrated an increased risk of 30-day readmission (p < 0.05) (Table 1). Operative factors, including operative time, elective status, or emergency status, were not associated with an increased readmission rate (p > 0.05). The patients who were readmitted had a significantly longer length of hospitalization (3.82 ± 0.12 vs. 4.65 ± 0.38, p = 0.02) and were less likely to have been discharged home (80.2% vs. 72.8%, p = 0.035). On multivariate analysis, preoperative hypertension, dialysis, sepsis, and ASA class >2 were statistically significant predictors of readmission following laminoplasty (Table 2). Of these, sepsis was the strongest predictor (OR = 5.58, 95% CI = 1.17-26.6, p = 0.03), followed by dialysis (OR = 3.46, 95% CI = 1.33-8.97, p = 0.01), ASA class >2 (OR = 1.69, 95% CI = 1.13-2.54, p = 0.011), and hypertension (OR = 1.51, 95% CI = 1.03-2.33, p = 0.04).

**Table 1:** Univariate analysis between non-readmitted versus readmitted in all cervical laminoplasty patients. Variables are listed with non-admitted patients totaling 2,938 and readmitted totaling 147. The mean with standard error, number of patients with percentage of total, and p-values are listed. Significant variables with p < 0.05 are bolded. BMI = body mass index; COPD = chronic obstructive pulmonary disease; CHF = congestive heart failure; ASA = American Society of Anesthesiologists

Variables	Non-readmitted (N = 2,938)	Readmitted (N = 147)	P-value
BMI	29.2 ± 0.14	30.0 ± 0.59	0.08
Operation time	162.79 ± 1.33	155.43 ± 5.40	0.09
Total length of stay	3.82 ± 0.12	4.65 ± 0.38	0.02
Preoperative duration	0.51 ± 0.04	0.71 ± 0.18	0.13
Male sex	1,901 (64.7%)	96 (65.3%)	0.93
Race			
White	1,920 (65.4%)	94 (63.9%)	0.84
Black	435 (14.8%)	25 (17.0%)	
Asian	258 (8.8%)	14 (9.5%)	
Native American	14 (0.5%)	0 (0.0%)	
Unknown	310 (10.6%)	14 (9.5%)	
Geriatric Age (≥65)	1,277 (43.5%)	76 (51.7%)	0.051
Home discharge	2,353 (80.2%)	107 (72.8%)	0.035
Elective surgery	2,577 (87.9%)	122 (83.6%)	0.12
Diabetes	663 (22.6%)	44 (29.9%)	0.044
Current smoker	593 (20.2%)	28 (19.0%)	0.83
Dyspnea	103 (3.5%)	9 (6.1%)	0.11
Independent status	2,769 (94.2%)	131 (89.1%)	0.019
COPD	129 (4.4%)	9 (6.1%)	0.31
CHF	20 (0.7%)	3 (2.0%)	0.09
Hypertension	1,653 (56.3%)	103 (70.1%)	0.001
Renal failure	6 (0.2%)	2 (1.4%)	0.052
Dialysis	25 (0.9%)	6 (4.1%)	0.003
Cancer	9 (0.3%)	0 (0.0%)	1.00
Chronic steroid use	111 (3.8%)	5 (3.4%)	1.00
Sepsis/Septic shock	7 (0.2%)	3 (2.0%)	0.01
Emergency surgery	64 (2.2%)	7 (4.8%)	0.08
Clean wound class	2,914 (99.2%)	143 (97.3%)	0.04
ASA class >2	1,691 (57.6%)	109 (72.1%)	<0.001

**Table 2:** Multivariate analysis between non-readmitted versus readmitted in all cervical laminoplasty patients. Variables included for regression analysis are listed with associated odds ratio and 95% confidence intervals and p-values. ASA = American Society of Anesthesiologists

Variables	Odds ratio	95% confidence interval	P-value
Total length of stay	0.99	0.96-1.02	0.55
Home discharge	0.91	0.60-1.39	0.67
Diabetes	1.069	0.73-1.58	0.74
Independent status	0.68	0.38-1.22	0.20
Hypertension	1.51	1.03-2.23	0.04
Dialysis	3.46	1.33-8.97	0.01
Sepsis/Septic shock	5.58	1.17-26.6	0.03
Clean wound class	0.54	0.16-1.83	0.33
ASA class >2	1.69	1.13-2.54	0.011

A subgroup analysis was performed of all geriatric patients (aged >65 years). In total, 1,353 patients were identified, and 76 were readmitted, demonstrating a readmission rate of 5.62%. On the univariate analysis baseline, demographic factors were not associated with readmission (p > 0.05) (Table 3). For preoperative comorbidities, hypertension, independent status, and ASA class >2 were statistically significantly associated with readmission (p < 0.05). For operative factors, surgery class and operative time were not associated with an increased risk of readmission. The total length of stay was statistically significantly longer in patients requiring readmission (5.42 ± 0.62 days vs. 3.98 ± 0.20 days, p = 0.03). On multivariate analysis, hypertension and independent status were statistically significant, with an OR of 1.98 (95% CI = 1.04-3.75, p = 0.004) and 0.39 (95% CI = 0.21-0.74, p = 0.04), respectively (Table 4).

**Table 3:** Univariate analysis between non-readmitted versus readmitted in geriatric cervical laminoplasty patients. Variables are listed with elderly (aged >65 years) non-admitted patients totaling 1,277 and readmitted totaling 76. The mean with standard error, number of patients with percentage of the total, and p-values are listed. Significant variables with p < 0.05 are bolded. BMI = body mass index; COPD = chronic obstructive pulmonary disease; CHF = congestive heart failure; ASA = American Society of Anesthesiologists

Variables	Non-readmitted (N = 1,277)	Readmitted (N = 76)	P-value
BMI	28.3 ± 0.19	29.1 ± 0.84	0.36
Operation time	157.62 ± 1.83	158.58 ± 8.54	0.91
Total length of stay	3.98 ± 0.20	5.42 ± 0.62	0.03
Preoperative duration	0.58 ± 0.06	0.74 ± 0.26	0.57
Male sex	793 (62.1%)	49 (64.5%)	0.72
Race			
White	874 (68.4%)	52 (68.4%)	0.87
Black	147 (11.5%)	9 (11.8%)	
Asian	118 (9.2%)	9 (11.8%)	
Native American	5 (0.4%)	0 (0.0%)	
Unknown	133 (10.4%)	6 (7.9%)	
Home discharge	900 (70.7%)	49 (64.5%)	0.25
Elective surgery	1,110 (87.2%)	63 (84.0%)	0.38
Diabetes	367 (28.7%)	27 (35.5%)	0.24
Current smoker	133 (10.4%)	7 (9.2%)	0.85
Dyspnea	54 (4.2%)	7 (9.2%)	0.08
Independent status	1,187 (93.0%)	62 (81.6%)	0.01
COPD	72 (5.6%)	6 (7.9%)	0.44
CHF	12 (0.9%)	2 (2.6%)	0.18
Hypertension	905 (70.9%)	64 (84.2%)	0.01
Renal failure	5 (0.4%)	2 (2.6%)	0.054
Dialysis	16 (1.3%)	3 (3.9%)	0.09
Cancer	4 (0.3%)	0 (0.0%)	1.00
Chronic steroid use	62 (4.9%)	1 (1.3%)	0.26
Sepsis/Septic shock	4 (0.3%)	1 (1.3%)	0.25
Emergency surgery	40 (3.1%)	4 (5.3%)	0.31
Clean wound class	1,265 (99.1%)	75 (98.7%)	0.53
ASA class >2	877 (68.7%)	62 (81.6%)	0.02

**Table 4:** Multivariate analysis between non-readmitted versus readmitted in geriatric cervical laminoplasty patients. Variables included for regression analysis are listed with associated odds ratio and 95% confidence intervals and p-values. ASA = American Society of Anesthesiologists

Variables	Odds ratio	95% confidence interval	P-value
Total length of stay	1.02	0.985-1.065	0.23
Independent status	0.39	0.21-0.74	0.004
Hypertension	1.98	1.04-3.75	0.04
ASA class >2	1.55	0.84-2.86	0.16

## Discussion

In this study, we examined the predictive factors of 3,085 patients who underwent cervical laminoplasty from 2011 to 2020. We examined patient demographics and preoperative, intraoperative, and postoperative variables associated with 30-day readmission in the general patient population and the elderly. We found that hypertension, sepsis, and ASA class >2 were predictive of readmission. In the elderly, independent status and hypertension were predictive. Sepsis during admission is the most predictive factor for readmission, which is expected as surgical site infections and other perioperative infections require re-evaluation, often resulting in readmission and surgical debridement and washout [10,11]. In this study, the rate of sepsis was 0.3%, which is consistent with previous literature [3,10,11]. Factors affecting surgical site infections include nutrition optimization and blood cell counts such as hematocrit and platelet levels [11]. Obesity with BMI >35 kg/m^2^ and chronic steroid use may also increase surgical site infections and are commonly used for risk stratification preoperatively [11]. Furthermore, operative efficiency may also reduce surgical site infections [11]. Although sepsis was the most significant factor contributing to readmission, several patient factors led to an increased risk of readmission.

Aside from infection and sepsis, this study suggests that patient comorbidities significantly impact the risk of readmission for patients undergoing cervical laminoplasty. This is first globally evidenced by an ASA class >2 being predictive of readmission in our study. This classification assesses surgical risk with classes 3 and 4 indicating severe systemic and severe systemic diseases that constantly threaten life. Previous literature found this classification to increase the length of stay in patients who underwent cervical spine procedures [9]. Passias et al. described the ASA score as a measurement of comorbid severity associated with worse outcomes, including the rate of readmission and complications [9]. Unfortunately, this factor may not be effectively mitigated within a reasonable preoperative period due to diseases that contribute to the score, such as chronic kidney disease (CKD), atherosclerosis, and other non-reversible diseases [9]. This is also highlighted in our study as dialysis status and hypertension were independent risk factors for readmission. Both may have related mechanisms for increasing the risk of readmission.

Dialysis status and vascular diseases have been implicated in increasing the risk of readmission after spine surgery. Sakaura et al. observed that dialysis status was associated with a higher risk of readmissions in posterior lumbar interbody fusion and laminoplasty [12]. In this study, CKD and systemic atherosclerosis were also associated with poor clinical outcomes, with atherosclerosis being an independent risk factor [12]. Thus, there appears to be a close relationship between vascular diseases and impaired renal function that contribute to a higher risk of readmission. We hypothesize that this relationship is why hypertension in our study was predictive of readmission. Although the relationship between preoperative hypertension and readmission rate in spine surgery has not been well studied, some orthopedic studies hypothesize preoperative hypertension interferes with wound healing and leads to infections [13,14]. Ahmed et al. demonstrated that hypertension was a risk factor for prolonged wound healing in total hip replacement surgery, while Yang et al. reported that hypertension led to a high risk of surgical site infection in orthopedic patients [13,14]. Thus, CKD and hypertension seem to negatively affect postoperative recovery and increase the risk of readmission.

Focusing on the elderly, independence status and hypertension were predictive factors for the elderly in readmission after cervical laminoplasty. Elderly patients may be at a higher risk of hypertension due to accumulated systemic disease and changes in physiology due to aging [15]. This may lead to an increased risk of readmission due to poor wound healing, but the elderly have a greater degree of risk due to their declining immune physiology and deconditioning. This is evidenced by the lack of independent status being a predictor of readmission. Impairment of independent activities of daily living (IADL) has been strongly associated with delirium, deconditioning, and stress, leading to infections, malnutrition, and polypharmacy, which contribute to the increased readmission rate [16]. These factors are especially devastating to recovery in the elderly, which is possibly why it was predictive only in elderly patients undergoing cervical laminoplasty. Examining patients with spinal cord injuries in eastern Massachusetts, an independent factor associated with lower length of stay was leaving home at least once daily, demonstrating the degree of impact activity can have on those who have impaired activity [17]. Therefore, extra vigilance should be practiced when operating on patients who are elderly and those with impaired IDAL as they are a factor that can be optimized before discharge, unlike chronic diseases [18]. Therefore, more careful attention to activity should be made in discharge and earlier therapy with ambulation during hospital stay after cervical laminoplasty.

There are several limitations to this study. The ACS-NSQIP database is limited as it does not record spine surgery-specific variables such as spine stability, alignment status, type of laminoplasty performed, and other anatomic features relevant to surgical risk stratification and procedural difficulty. This may be mitigated by analyzing operative time, which grossly estimates risk and anatomic difficulty but is only a surrogate estimation. Furthermore, the database only records data for the 30-day postoperative period, which misses long-term complications and admissions relating to the surgery beyond this timeframe.

## Conclusions

Evaluation of predictive factors for readmission is important for patient education and the setting of appropriate clinical expectations for surgeons and providers. Preoperative hypertension, global comorbid status, and renal function were independent risk factors for 30-day readmission following cervical laminoplasty, with the independent status being a unique predictor among the elderly. Although many of these factors are difficult to mitigate within the timeframe of surgery, promoting activity and independence, which can be done prior to discharge, may reduce readmission risk, especially in elderly patients.
